# Supplementary material for: Enhanced Antitumor Efficacy of PhAc-ALGP-Dox, an Enzyme-Activated Doxorubicin Prodrug, in a Panel of THOP1-Expressing Patient-Derived Xenografts of Soft Tissue Sarcoma
Source: Biomedicines. 2022 Apr 6;10(4):862. doi: 10.3390/biomedicines10040862 (PMC9025547; doi:10.3390/biomedicines10040862)
Supplement: Supplementary file 1 [file biomedicines-10-00862-s001.zip › biomedicines-1628987-SM-xml(1).pdf]

Supplementary Material

Table S1. Thimet oligopeptidase (THOP1) expression and tumor growth delay in the patient-derived sarcoma xenografts as determined by enzyme-linked immunoassay (ELISA) and immunohistochemistry (IHC).

Xenografts	THOP1 IHC SCORE	THOP1 ELISA (ng/g total protein)	Tumor Growth Delay (day 29)	
			**CBR-049**	**CBR-050**
UZLX-STS84^{UPS}	0	22.67 ± 3.15	52.95	29.28
UZLX-STS216^{MFS}	1	143.27 ± 50.82	77.65	n/d
UZLX-STS234^{eOS}	1	197.60 ± 37.26	47.39	n/d
UZLX-STS211A^{UPS}	1	224.04 ± 59.37	59.72	n/d
UZLX-STS22^{LMS}	2	26.84 ± 1.80	71.34	10.81
UZLX-STS7^{SynSa}	2	74.81 ± 13.55	75.75	31.38
UZLX-STS128^{LMS}	2	203.86 ± 22.06	81.89	15.46
UZLX-STS204^{DDLPS}	2	299.36 ± 6.38	78.01	n/d
UZLX-STS89^{MFS}	3	29.98 ± 8.04	94.78	87.24
UZLX-STS185^{IS}	3	171.31 ± 23.71	86.39	59.57

DDLPS: dedifferentiated liposarcoma; eOS: extraskeletal osteosarcoma; IHC: immunohistochemistry; IS: intimal sarcoma; LMS: leiomyoscarcoma; MFS: myxofibrosarcoma; n/d: not done; SynSa: synovial sarcoma; UPS: undifferentiated pleomorphic sarcoma; 0: negative; 1: weakly positive; 2: intermediate positive; 3: strongly positive.

Table S2. Detailed description on the number of mice/tumors included in each experiment. Data represent number of mice/tumors at begin of the experiment (day 1) and number mice/tumors at the day of statistical analysis (day 36/day 29) between brackets.

Xenografts	Control	Doxo	CBR-049	CBR-050	Aldoxo
UZLX-STS7^{SynsSa}	12 (4)	12 (11)	10 (9)	8 (4)	n/d
UZLX-STS22^{LMS}	7 (3)	7 (4)	8 (6)	8 (4)	n/d
UZLX-STS84^{UPS}	9 (4)	9 (8)	10 (6)	10 (3)	n/d
UZLX-STS89^{MFS}	11 (9)	11 (11)	11 (10)	11 (10)	n/d
UZLX-STS128^{LMS}	10 (8)	10 (9)	10 (9)	10 (7)	n/d
UZLX-STS185^{IS}	9 (5)	9 (8)	10 (8)	10 (7)	n/d
UZLX-STS204^{DDLPS}	10 (9)	12 (12)	12 (12)	n/d	12 (12)
UZLX-STS211A^{UPS}	10 (10)	12 (12)	12 (11)	n/d	12 (12)
UZLX-STS216^{MFS}	10 (10)	12 (11)	12 (12)	n/d	12 (9)
UZLX-STS234^{eOS}	10 (10)	11 (11)	12 (12)	n/d	11 (7)

DDLPS: dedifferentiated liposarcoma; eOS: extraskeletal osteosarcoma; IS: intimal sarcoma; LMS: leiomyoscarcoma; MFS: myxofibrosarcoma; n/d: not done; SynSa: synovial sarcoma; UPS: undifferentiated pleomorphic sarcoma.

Table S3. Relative tumor volumes compared to baseline (%) at the day of statistical analysis presented as average ± standard deviation. DDLPS: dedifferentiated liposarcoma; eOS: extraskeletal osteosarcoma; IS: intimal sarcoma; LMS: leiomyosarcoma; MFS: myxofibrosarcoma; n/d: not done; SynSa: synovial sarcoma; UPS: undifferentiated pleomorphic sarcoma; * $p < 0.05$ compared to control; # $p < 0.05$ compared to doxorubicin (doxo); ** $p < 0.005$ compared to control; ## $p < 0.005$ compared to doxo; *** $p < 0.0005$ compared to control; ### $p < 0.0005$ compared to doxo.

	Xenograft model	Control	Doxo	CBR-049	CBR-050	Aldoxo
Day 36	UZLX-STS7^{SynSa}	1801 ± 1269	135 ± 108**	187 ± 235*	998 ± 317$^{\#\#}$	n/d
	UZLX-STS22^{LMS}	1098 ± 824	962 ± 542	190 ± 28*$^{\#}$	604 ± 275	n/d
	UZLX-STS84^{UPS}	2800 ± 1398	1579 ± 1087	486 ± 503*$^{\#}$	1799 ± 130	n/d
	UZLX-STS89^{MFS}	898 ± 556	154 ± 190**	51 ± 20***$^{\#\#}$	238 ± 249**	n/d
	UZLX-STS128^{LMS}	775 ± 338	508 ± 154	62 ± 43***$^{\#\#\#}$	661 ± 389	n/d
	UZLX-STS185^{IS}	2804 ± 984	938 ± 381**	444 ± 252**$^{\#\#}$	1340 ± 822	n/d
Day 29	UZLX-STS204^{DDLPS}	559 ± 350	276 ± 162	123 ± 43**$^{\#\#}$	n/d	125 ± 49**$^{\#\#}$
	UZLX-STS211A^{UPS}	657 ± 275	502 ± 209*	265 ± 90**$^{\#\#}$	n/d	275 ± 119**$^{\#\#}$
	UZLX-STS216^{MFS}	786 ± 569	491 ± 333	176 ± 242**$^{\#\#}$	n/d	82 ± 54***$^{\#\#\#\#}$
	UZLX-STS234^{eOS}	453 ± 219	451 ± 284	238 ± 79**$^{\#}$	n/d	257 ± 72*$^{\#}$

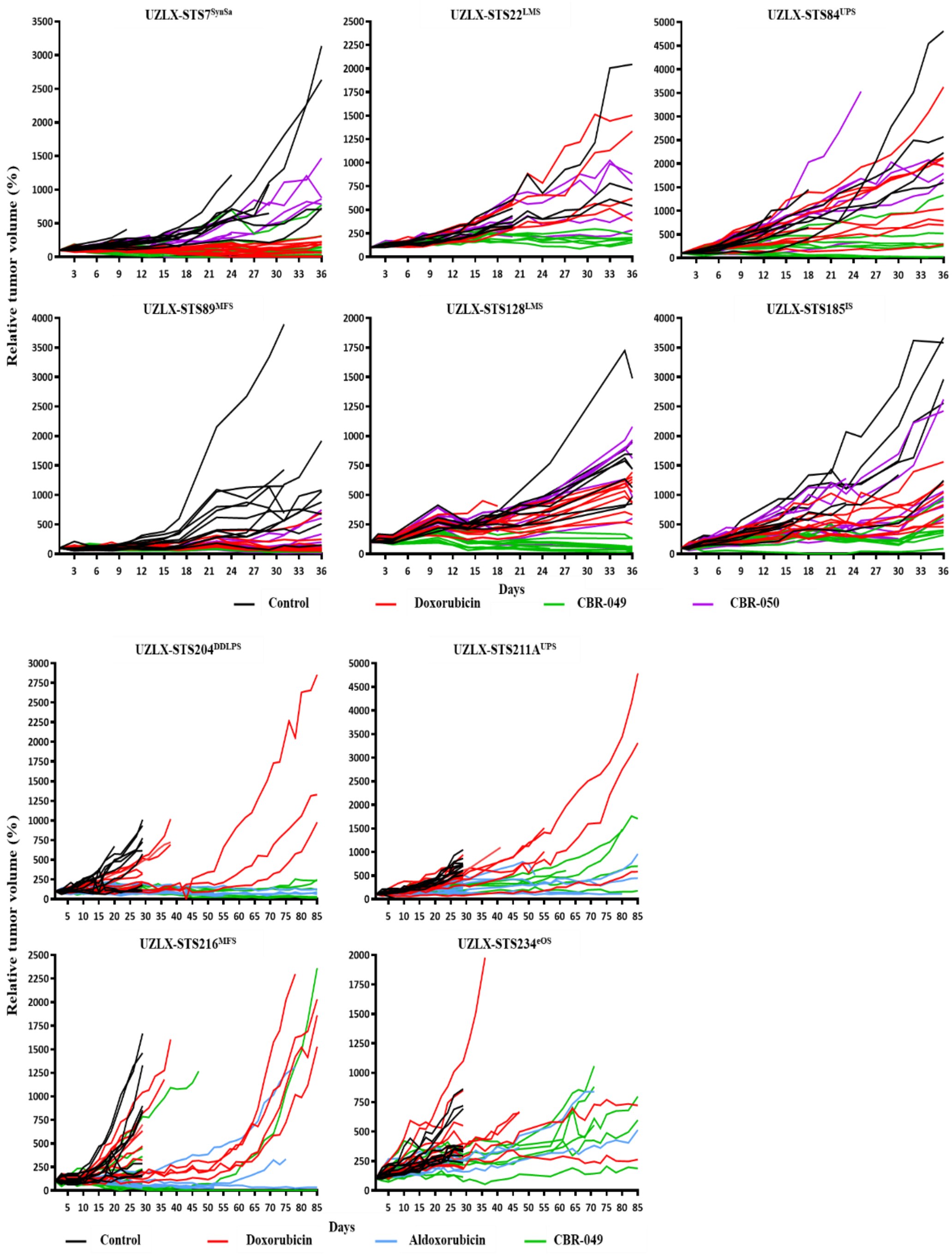

Figure S1. Individual relative tumor growth curves of all ten xenograft experiments.

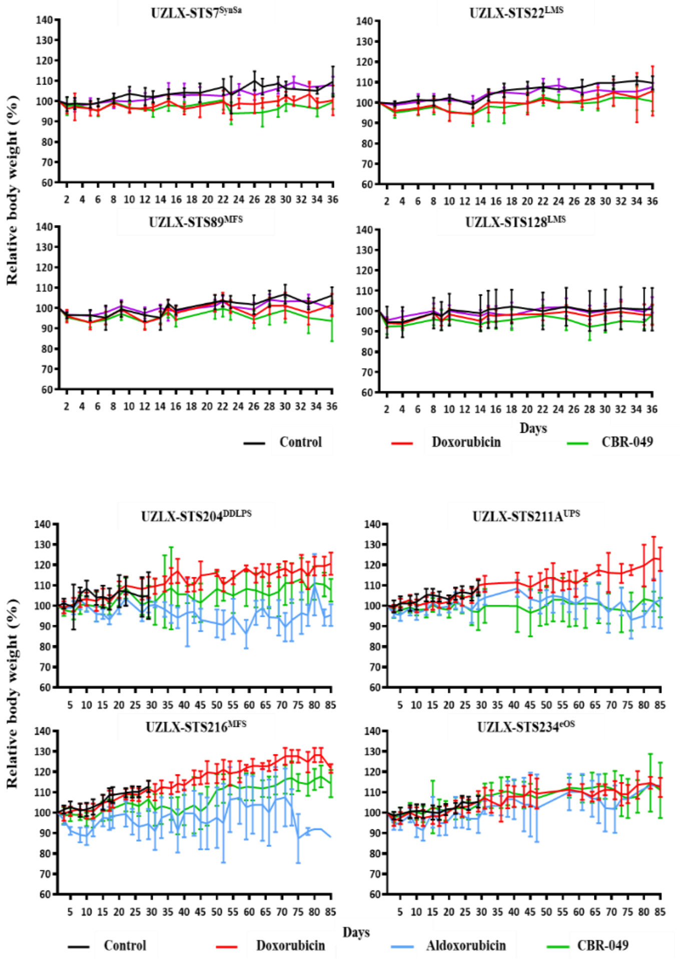

Figure S2. Relative body weight (%) compared to baseline in all ten patient-derived sarcoma xenografts included in this study, presented as average ± standard deviation.
